# Laparoscopic and Endoscopic Cooperative Surgery for Gastric Cancer as an Alternative Treatment in Elderly Patients: A Prospective Observational Study

**DOI:** 10.1002/ags3.70108

**Published:** 2025-10-29

**Authors:** Marie Washio, Keishi Yamashita, Takuya Wada, Mikiko Sakuraya, Hiroki Harada, Tadashi Higuchi, Koshi Kumagai, Yasushi Orihashi, Chika Kusano, Naoki Hiki

**Affiliations:** ^1^ Department of Upper Gastrointestinal Surgery Kitasato University School of Medicine Sagamihara Kanagawa Japan; ^2^ Research and Development Center for Medical Education, Department of Clinical Skills Education Kitasato University School of Medicine Sagamihara Kanagawa Japan; ^3^ Division of Advanced Surgical Oncology, Department of Research and Development Center for New Medical Frontiers Kitasato University School of Medicine Sagamihara Kanagawa Japan; ^4^ Department of Gastroenterology Kitasato University School of Medicine Sagamihara Kanagawa Japan; ^5^ Clinical Research Promotion Center Kitasato University Hospital Sagamihara Kanagawa Japan; ^6^ Clinical Research Center in Hiroshima Hiroshima University Hospital Hiroshima Japan

**Keywords:** elderly gastric cancer, laparoscopic and endoscopic cooperative surgery, minimally invasive surgery, palliative surgery, quality of life

## Abstract

**Aim:**

Laparoscopic and endoscopic cooperative surgery (LECS) is a minimally invasive procedure that preserves gastric function and reduces complications. Although widely applied for gastrointestinal stromal tumors, its role in gastric cancer remains uncertain. We prospectively evaluated LECS as an alternative for elderly gastric cancer patients who declined conventional gastrectomy.

**Methods:**

This single‐center prospective study enrolled 20 patients aged 75–89 years with gastric cancer ≤ 5 cm. All patients underwent Inverted‐LECS using the Crown method. The primary endpoint was surgery‐related morbidity (Clavien–Dindo ≥ III). Secondary endpoints were operative outcomes, margin status, quality of life (QOL), and survival.

**Results:**

Median age was 82 years, and 80% were cT1. No intraoperative complications or conversions occurred. Surgical morbidity ≥ Grade III was 0%. Delayed gastric emptying occurred in 25%, all Grade II. Resection margins were negative in 80%, including all pathologically early cancers. At 3 months, body weight and fat‐free mass were preserved, physical function scores recovered to baseline, and mental function improved. Ninety‐day mortality was 0%. With a median follow‐up of 32 months, disease progression occurred in six patients, mainly with advanced disease or positive margins. One‐ and 3‐year overall survival rates were 95% and 76.2%, and disease‐specific survival 95% and 90%.

**Conclusions:**

LECS was technically safe and feasible for elderly gastric cancer patients ineligible for gastrectomy. However, because oncological curability cannot be assured in advanced gastric cancer or high‐grade lymph node metastasis, careful selection and informed consent are essential. LECS may serve as a functional‐preserving alternative to observation or palliative treatment.

## Graphical abstract

In a prospective study of 20 elderly gastric cancer patients who declined gastrectomy, laparoscopic and endoscopic cooperative surgery (LECS) was technically safe and preserved postoperative function. However, as oncological curability cannot be assured in advanced cases, careful selection and informed consent are essential.

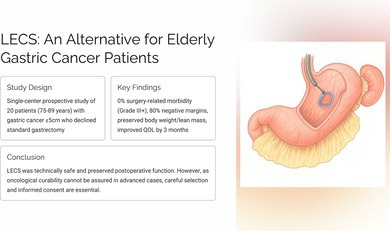

## 1 Introduction

Laparoscopic and endoscopic cooperative surgery (LECS) was first introduced in 2006 for local resection of gastric submucosal tumors (SMTs) [1]. In 2014, LECS was approved as an insured procedure, termed Classical LECS, and became the standard approach for gastric local resection in Japan. The most important feature of LECS is its ability to achieve minimal gastric resection while preserving blood flow and nerve supply, thereby reducing postoperative complications and preventing decreased food intake and post‐gastrectomy disorders.

Classical LECS involves intentional gastric wall opening, which raises concerns about tumor cell dissemination in the presence of malignancy. To prevent tumor cell dissemination and enable LECS application for gastric cancer, various LECS‐related techniques have been developed, including inverted LECS, nonexposed endoscopic wall‐inversion surgery (NEWS), closed LECS, and the combination of laparoscopic and endoscopic approaches to neoplasia with a non‐exposure technique (CLEAN‐NET). These LECS‐related techniques prevented intraperitoneal tumor cell scattering, allowing LECS to be performed safely for malignant tumors [2, 3]. Consequently, LECS was covered by insurance in Japan as a procedure for gastric cancer in 2020.

As Japan's population continues to age, the proportion of elderly patients with gastric cancer is increasing [4, 5]. Many elderly patients are at increased risk of postoperative complications following conventional gastrectomy due to comorbidities [6, 7, 8]. Additionally, it is well known that some patients experience a significant decline in quality of life (QOL) due to weight loss from reduced food intake post‐gastrectomy [9].

Given these factors, some elderly patients or those with severe comorbidities may refuse extensive gastrectomy, even when they understand that it is required for curative treatment. In such cases, several alternative treatments may be considered. One is observation without active intervention, which carries the risk of tumor‐related bleeding or obstruction. As established palliative treatments, gastrojejunostomy can be used to relieve obstruction, and radiotherapy can be applied to control bleeding; however, both are susceptible to tumor progression, potentially leading to recurrent symptoms.

In contrast, local resection using LECS may offer more definitive control of both current and anticipated symptoms. Although its role in gastric cancer remains unclear, LECS may be a promising alternative for selected patients.

To explore its potential, we conducted a prospective observational study to evaluate the efficacy and safety of LECS as an alternative treatment for local disease control in gastric cancer. The reasons for selecting LECS varied and included refusal of standard gastrectomy, high perioperative risk, and clinical frailty. All patients provided informed consent, acknowledging the non‐curative intent of the procedure.

## 2 Methods

### 2.1 Patient Recruitment

This prospective, single‐center, single‐arm trial included patients aged 75–89 years with histologically confirmed gastric cancer who underwent LECS. Eligibility criteria included patient‐ and disease‐related factors. Patient‐related criteria included as follows: (1) sufficient organ function for general anesthesia and (2) the ability to maintain oral intake. Disease‐related criteria included as follows: (1) tumor diameter ≤ 5 cm; (2) no absolute indication for ESD; (3) macroscopic type other than Borrmann type 4; (4) no invasion of the esophagus or duodenum; (5) no prior gastric cancer surgery; and (6) no evidence of multiple distant peritoneal metastases on CT. Tumors involving both the anterior and posterior walls of the gastric antrum were excluded due to the risk of severe gastric peristalsis from Latarjet nerve transection. Patients with psychosis or other psychiatric disorders were also excluded. Among eligible patients, those who declined standard curative gastrectomy with lymph node dissection—despite sufficient explanation based on the Japanese Gastric Cancer Treatment Guidelines—were offered LECS as an alternative.

### 2.2 Operative Procedure of LECS as an Alternative Treatment for Gastric Cancer

#### 2.2.1 Determination of Resection Line

The extent of resection was determined based on tumor type. For early‐stage cancers, multiple biopsies were taken from the periphery of the lesion to confirm the absence of cancer cells, and the resection line was planned according to ESD criteria. For advanced gastric cancers, the resection area was planned to remain within approximately 8 cm, with at least a 5 mm margin from the tumor edge in localized cases. LECS was also performed for infiltrative‐type cancers when strongly requested by the patient, but only in cases where the tumor was considered resectable within this size range, although an exact margin could not be clearly defined due to the unclear tumor boundaries.

#### 2.2.2 Surgical Procedure

**FIGURE 1:**
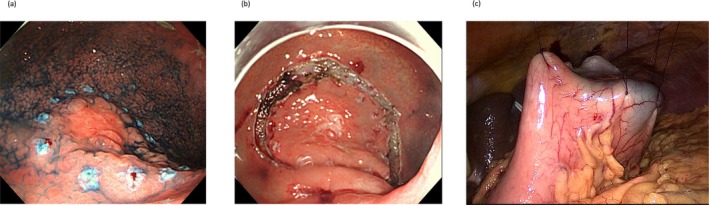
Key steps of the Inverted‐LECS procedure using the crown method. (a) Coagulation markings are placed around the tumor. (b) Circumferential incision is made down to the submucosal layer. (c) Traction sutures are placed on the gastric wall and pulled ventrally toward the abdominal wall, thus forming a crown‐like shape.

We previously reported the inverted‐LECS procedure using the crown method for gastric cancer, and the same technique was applied in this study (Figure 1) [10]. After performing a circumferential submucosal incision, the vessels within the resection area were dissected, and sutures were placed around the edges of the gastric incision to lift the stomach wall ventrally, forming a crown‐like shape. The tumor was resected endoscopically and dropped into the gastric lumen to prevent contact with intra‐abdominal tissues.

The approach to lymph nodes during surgery was intentionally left undefined in this study. Lymph nodes attached to the gastric wall within the resection area—such as stations No. 1, 3, 4sb, and 4d—were removed along with the specimen.

Most tumors were retrieved via an oral endoscope; however, when this was not feasible, the tumors were placed in plastic bags and removed from the abdominal cavity. The gastric wall defect was closed laparoscopically, either by hand‐sewing or with a laparoscopic stapling device following temporary suturing.

### 2.3 Adjuvant Treatment Policy

As lymph node dissection was not performed in this study, precise pathological staging could not be determined, making it impossible to apply evidence‐based chemotherapy. Therefore, the protocol‐defined treatment consisted of surgery alone, and the protocol was considered complete upon surgical completion. Postoperative treatment, including chemotherapy, was left to the discretion of the patient and the treating physician, as it was not part of the protocol‐defined treatment.

### 2.4 Follow‐Up

Postoperative follow‐up was conducted based on a fixed schedule, as summarized in Table S1. Routine assessments—including tumor marker monitoring, endoscopy, chest radiography, and abdominal CT—were planned through the postoperative surveillance period.

Body composition was evaluated using the InBODY S10 (InBody Co. Ltd., Seoul, Korea) at regular intervals during the first year and annually thereafter, measuring body weight (BW) and lean body mass (LBM). Postoperative QOL was assessed using the Postgastrectomy Syndrome Assessment Scale‐45 (PGSAS‐45) at 1 and 3 months, and at 1, 2, and 3 years after surgery. The PGSAS‐45 is a validated instrument comprising 37 items on postgastrectomy symptoms and 8 items from the Short Form‐8 Health Survey (SF‐8), which evaluate general physical and mental health [11, 12, 13]. The SF‐8 yields two summary scores: the physical component summary (PCS) and mental component summary (MCS).

### 2.5 Endpoints

The primary endpoint was the incidence of “surgery‐related morbidity” classified as Clavien–Dindo classification (C–D) Grade III or higher within 1 month postoperatively [14]. Secondary endpoints included operation time, estimated blood loss, length of postoperative hospital stay, cancer‐negative margin rate, BW, LBM, QOL assessment by PGSAS‐45, local recurrence rate, overall survival (OS), and disease‐specific survival (DSS).

### 2.6 Statistical Analysis

Based on the retrospective data, the proportion of postoperative complications (C–D Grade III or higher) in elderly gastric cancer patients ranged from 7.2% to 16.7% [6, 7, 8]. Assuming a 5% incidence of “surgery‐related complications” for the proposed surgical procedure, a sample size of 47 patients was calculated to be necessary to demonstrate, with 80% power, that the incidence rate would be less than 16.7% at a one‐sided significance level of 5%. The final sample size was set at 50 patients. However, due to slower than expected patient accrual, the study was concluded after enrolling 20 patients. Given the exploratory and observational nature of the study, the statistical analysis plan was not modified, and data were analyzed and reported as originally planned. This change was approved by the institutional review board, and the study was continued as a prospective observational study.

Baseline characteristics, surgical outcomes, and pathological findings were summarized as medians and ranges for continuous variables and as counts and percentages for categorical variables. The numbers and percentages of patients with postoperative complications were calculated. One‐sided 95% confidence limits (equivalent to two‐sided 90% confidence intervals) for the proportion of postoperative complications were calculated based on Wilson confidence limits (score confidence limits). Scores for BW and LBM, as well as QOL scores assessed using the PGSAS‐45, were summarized as medians and ranges. Values at 1 and 3 months postoperatively were compared using paired *t*‐tests. Similarly, scores for the PCS and MCS were summarized as medians and ranges, while values at the 1‐month and 3‐month time points were compared with those at the preoperative time point. All *p* values were two‐sided, and *p* < 0.05 indicated statistical significance.

All statistical analyses were performed using JMP for Windows, version 18 (SAS Institute Inc., Cary, NC, USA).

## 3 Results

### 3.1 Patients' Background

Between April 2020 and February 2024, 20 patients were enrolled in the study and treated with LECS. Patient characteristics are presented in Table 1. The median age was 82 (78–88) years. Eastern Cooperative Oncology Group (ECOG) performance status was 0 in 18 patients and 1 in 2 patients. All 20 patients had comorbidities classified as American Society of Anesthesiologists Physical Status Classification System (ASA‐PS) 2 or higher, with 4 patients (20%) classified as ASA‐PS 3. The median tumor diameter was 3.3 (1–5) cm. Sixteen patients (80%) had tumors with a depth of cT1, and 18 patients (90%) had no clinical lymph node metastasis.

**TABLE 1:** Baseline characteristics of all enrolled patients.

	(*n* = 20)
Age, median (range)	82 (78–88)
Gender (male/female)	16/4
Body mass index, median (range)	23.2 (18.1–30.3)
ECOG performance status (0/1)	18/2
ASA‐PS (Class II/III)	16/4
Primary tumor location (upper third/middle third/lower third)	9/8/3
Primary tumor location (ante/post/less/gre)	1/11/5/3
Clinical maximum diameter (cm), median (range)	3.3 (1–5)
cT1a/T1b/T2/T3/T4a	3/13/3/0/1
cN−/+	18/2
Histology (differentiated type/undifferentiated type)	13/7

Abbreviations: ASA‐PS, American Society of Anesthesiologists physical status classification system; ECOG, Eastern Cooperative Oncology Group.

### 3.2 Early Operative Outcome

Surgical findings and postoperative complications are summarized in Table 2. The median operative time was 312 (170–469) minutes, and the median estimated blood loss was 0 (0–300) mL. There were no intraoperative adverse events, and no cases required conversion to an open approach. The median postoperative hospital stay was 7 (3–26) days.

**TABLE 2:** Early operative outcome.

Surgical outcome		(*n* = 20)
Total operative time (min), median (range)	312	(170–469)
Estimated blood loss (mL), median (range)	0	(0–300)
Intraoperative adverse event, *n*	0	
Conversion to the open method, *n*	0	
Postoperative hospital stay (days), median (range)	7	(3–26)
Postoperative complications	CD I‐II, *n* (%)	CD III, *n* (%)
Surgery‐related morbidity		
Delayed gastric emptying	5 (25)	0
Arrythmia	1 (5)	0
Pneumonia	1 (5)	0
Intra‐abdominal infectious complications	0	0
(Suture failure, pancreatic fistula, abdominal abscess)		
Obstruction or ileus	0	0
Non‐surgery‐related morbidity		
Prostatic abscess	1 (5)	0
Infectious intervertebral discitis	1 (5)	0
Cardiac tamponade	0	1 (5)

Abbreviation: C–D, Clavien–Dindo classification.

Surgery‐related morbidity (C–D Grade III or higher) set as the primary endpoint occurred in 0 patients (0%). The 90% confidence interval was 0%–11.9%, significantly below the prespecified threshold of 16.7% (one‐sided *p* = 0.026), confirming that the primary endpoint was met. Overall, 8 patients (40%) had postoperative complications. Delayed gastric emptying (DGE) occurred in 5 patients, all of whom improved with medication (C–D Grade II). Postoperative pneumonia and cardiac tamponade occurred in 1 patient, who was treated with echo‐guided pericardial drainage for the tamponade (C–D Grade III). One patient developed postoperative arrhythmia (Sick Sinus Syndrome) that resolved without intervention (C–D Grade I). Another patient developed a prostatic abscess, which led to infectious intervertebral discitis, which was cured by long‐term antibiotic therapy and prolonged bed rest (Grade II).

There were no cases of intra‐abdominal infectious complications, such as suture failure, pancreatic fistula, or intra‐abdominal abscess. Furthermore, there were no cases of postoperative bowel obstruction. No patient required reoperation.

### 3.3 Pathological Findings

Pathological findings are summarized in Table 3. Tumor depth was classified as pT1a/T1b/T2/T3/T4a in 5/9/2/2/2 cases, respectively.

**TABLE 3:** Pathological findings.

	(*n* = 20)
Pathological maximum diameter (cm), median (range)	3 (1.2–7.5)
Histology (differentiated type/undifferentiated type)	13/7
pT1a/T1b/T2/T3/T4a	5/9/2/2/2
*Resected lymph nodes*	
Cases of positive lymph node metastasis, *n*	5
Number of metastatic lymph nodes (per patient)^†^	1 (*n* = 2), 2 (*n* = 1), 8 (*n* = 1), 9 (*n* = 1)
Margin (negative/positive)	16/4

^†^ Numbers in parentheses indicate the number of patients.

Lymphadenectomy was not performed in this study; however, lymph nodes resected as part of the full‐thickness excision around the tumor were evaluated histologically. As a result, lymph node removal was recorded in 11 patients, of whom 5 had positive lymph node metastases. Among these 11 patients, the number of removed lymph nodes ranged from 1 – 11, and the number of metastatic nodes per patient ranged from 0 – 9.

Resection margins were negative in 16 cases (80%). The four margin‐positive cases were all pathologically diagnosed as advanced gastric cancer (≥ pT2). One of them had been diagnosed as advanced cancer preoperatively, whereas the other three had been preoperatively classified as early gastric cancer, all with undifferentiated histology. Additional surgery was discussed with all four patients; however, none opted for further resection. Postoperative chemotherapy was not administered in any of the cases. Although postoperative treatment was left to the discretion of the attending physicians and patients, no one chose to receive additional systemic therapy, as all surgeries were performed with non‐curative intent. Three of these four margin‐positive patients were among the six who experienced disease progression, and their pathological details are included in Table 4.

**TABLE 4:** Details of disease progression cases.

Case	Macroscopic	Clinical T	Biopsy	Pathological T	Pathological N	Pathological histology	Margin	Progression site	Survival	Time (months)
1	Type 3	T4a	tub1	T4a	N3 (9/11)	por2	+	Local, LN, P	Death (gastric cancer)	13.9
2	Type 2	T1b	por	T2	Nx (0/0)	por2	+	LN	Death (other)	21.9
3	0–IIc	T1b	sig	T4a	Nx (0/0)	por2	+	P	Alive	32.2
4	Type 2	T2	tub2	T2	≥ N1 (2/4)	tub2	−	LN, Adrenal	Alive	40.6
5	Type 2	T2	tub1	T3	≥ N1 (1/4)	tub1	−	LN	Alive	14.5
6	0–III	T1b	por	T1b	N3 (8/8)	por1	−	LN, P, Liver	Death (gastric cancer)	5.5

*Note:* ≥ *N* category indicates the minimum number of metastatic lymph nodes identified, based on limited nodal sampling.

Abbreviations: LN, lymph node; margin+, positive resection margin; margin−, negative resection margin; Nx, not assessable due to limited lymph node sampling only; P, peritoneal.

### 3.4 Change in BW and LBM After LECS

**FIGURE 2:**
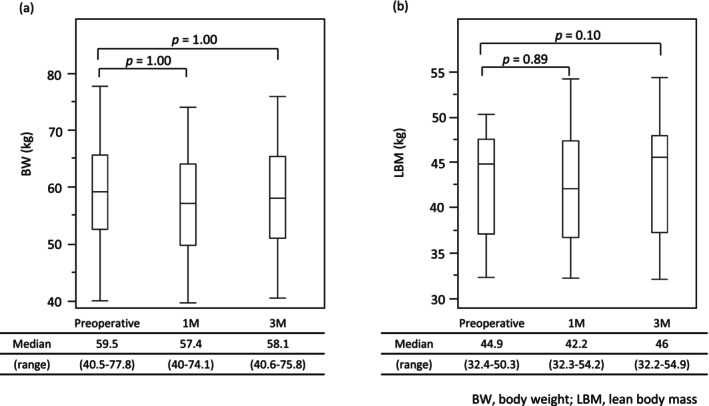
Changes in Body Weight and Lean Body Mass. (a) Body weight (BW) at 1 and 3 months postoperatively showed no significant differences compared to preoperative values. (b) Lean body mass (LBM) at 1 and 3 months postoperatively also showed no significant differences compared to preoperative values.

Changes in BW and LBM are presented in Figure 2. Both BW and LBM at 1 month and 3 months postoperatively showed no significant difference compared to preoperative values.

### 3.5 QOL After LECS

**FIGURE 3:**
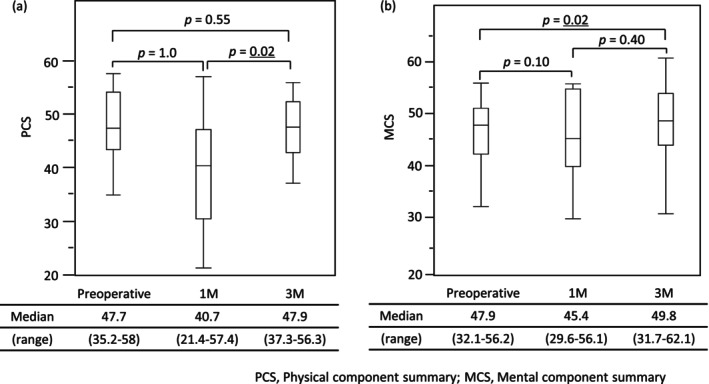
Trends in General Health. (a) The physical component summary (PCS) value tended to decrease at 1 month postoperatively, though not significantly, but improved significantly by 3 months, reaching levels equivalent to the preoperative value. (b) The mental component summary (MCS) value did not decrease at 1 month postoperatively and improved significantly by 3 months, exceeding the preoperative value.

QOL was assessed at 1 and 3 months postoperatively using the PGSAS‐45, as shown in Table S2. None of the parameters worsened at 3 months compared to 1 month. Significant improvements were observed in weight loss rate, food intake, and food quality at 3 months. Figure 3 shows the PCS and MCS values at the preoperative baseline, 1 month, and 3 months. The PCS value tended to decrease slightly at 1 month but improved significantly at 3 months, returning to the preoperative level. The MCS value did not decline at 1 month and was significantly higher than the preoperative value at 3 months.

### 3.6 Survival

**FIGURE 4:**
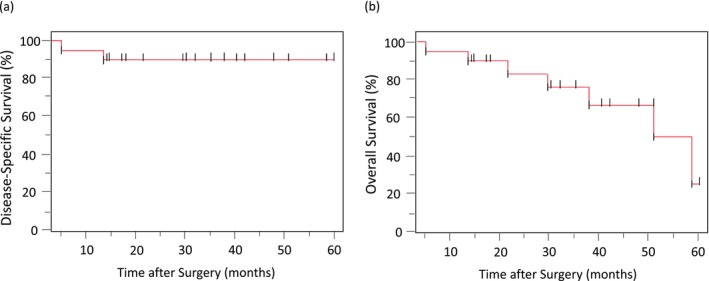
Kaplan–Meier curves for disease‐specific survival (DSS) and overall survival (OS). (a) DSS was defined as the time from surgery to death from gastric cancer. Deaths from other causes were censored in the DSS analysis. (b) OS was defined as the time from surgery to death from any cause. Censored observations are indicated by vertical ticks.

The median observation period was 32.2 months (range, 14.5–60). No deaths occurred within 3 months postoperatively. Kaplan–Meier curves for DSS and OS are shown in Figure 4. During the study period, 7 patients died, including 2 deaths due to gastric cancer and 5 deaths from non‐cancer‐related causes.

Disease progression after LECS was observed in 6 patients, and the details and outcomes of these cases are summarized in Table 4. Of these 6 patients, 3 had positive surgical margins. Sites of progression in these cases included local recurrence, lymph node metastasis, and peritoneal dissemination, with one patient dying of gastric cancer at 13.9 months postoperatively. The remaining margin‐positive case did not experience disease progression and died of a non‐cancer‐related cause at 38.0 months. In margin‐negative cases, the sites of progression included lymph nodes, peritoneum, adrenal gland, and liver. Among them, one patient, diagnosed as N3 based on resected lymph nodes, died of gastric cancer at 5.5 months postoperatively. None of the patients with disease progression received additional treatment, as all declined further therapy.

The 1‐year and 3‐year overall survival (OS) rates were 95% and 76.2%, respectively. The 1‐year and 3‐year disease‐specific survival (DSS) rates were 95% and 90%, respectively.

## 4 Discussion

LECS has demonstrated favorable short‐ and midterm outcomes in SMTs, particularly gastrointestinal stromal tumors (GISTs) [15, 16] However, no prospective studies to date have comprehensively evaluated its use in gastric cancer. In this study, we focused on elderly patients who declined standard curative gastrectomy and assessed the procedural safety of LECS as the primary endpoint. Notably, there were no surgery‐related deaths or C–D Grade III or higher complications directly attributable to the procedure.

The most common complication was delayed gastric emptying (DGE), observed in five patients (25%), all of whom were Grade II and resolved with conservative treatment. Although four of the five patients had hospital stays exceeding the overall median, none experienced prolonged morbidity or required invasive intervention. In addition to DGE, only one case of mild pneumonia (C–D Grade I) was observed. Another patient developed cardiac tamponade (C–D Grade III), which was deemed unrelated to the surgery. Importantly, no non‐cancer‐related deaths occurred within 90 days postoperatively. Of note, 60% of patients had a predicted perioperative mortality risk ≥ 1% according to the National Clinical Database risk calculator, suggesting that LECS may be a safe option even in high‐risk populations [17, 18].

This favorable complication profile may be partly due to surgical strategies designed to prevent DGE. Specifically, tumors requiring wide transection of the Latarjet nerve, which plays an essential role in gastric peristalsis, were excluded; tumor size was limited to ≤ 5 cm; and an oblique‐axis closure technique was employed to minimize gastric deformation and luminal narrowing. These measures likely helped mitigate severe DGE and reduce the risk of aspiration pneumonia. Furthermore, early recognition of DGE and timely initiation of supportive care—including temporary fasting—may have contributed to preventing progression and minimizing clinical impact [19].

In all cases, we used the Crown method, an open‐type LECS. Although non‐exposure techniques such as NEWS and CLEAN‐NET reduce the theoretical risk of peritoneal contamination, they were not technically feasible in the relatively large gastric cancers treated in this cohort. During follow‐up, peritoneal dissemination was observed in three patients. As all involved were advanced tumors, the direct impact of the open approach remains uncertain. While the Crown method has been safely applied to GISTs, including Delle‐positive lesions, its oncologic safety in epithelial malignancies remains uncertain, and long‐term follow‐up in larger patient populations is essential.

All pathologically confirmed early‐stage cancer cases achieved negative resection margins, and combined with the low complication rate, LECS may be considered a treatment alternative in patients ineligible for standard gastrectomy. However, the recurrence risk cannot be excluded, underscoring the need for extended surveillance. In the case of advanced cancer, LECS carries a higher likelihood of positive resection margins due to difficulties in achieving oncologically adequate local excision. Additionally, in clinically node‐positive (cN+) cases, the lack of lymphadenectomy may lead to inadequate disease control.

In this study, positive resection margins were identified in 4 of the 20 patients (20%). One of these patients had been diagnosed with advanced gastric cancer preoperatively; this individual presented with a Type 3 tumor and subsequently developed local recurrence accompanied by impaired gastric peristalsis, ultimately resulting in difficulty with oral intake. The remaining three cases had been clinically diagnosed as cT1 tumors but were pathologically confirmed as advanced gastric cancers with undifferentiated histology.

These findings underscore two important considerations for patient selection. First, when preoperative imaging or endoscopic findings strongly suggest advanced disease—particularly in diffuse‐type tumors or those with poorly defined margins—LECS should be avoided, as achieving negative margins may be difficult, even when the procedure is intended as an alternative treatment. Second, even in cases preoperatively diagnosed as early‐stage gastric cancer, undifferentiated‐type tumors may be pathologically upstaged following resection, increasing the risk of both positive margins and early recurrence. Therefore, particular caution is warranted when accurate preoperative assessment of tumor extent is challenging. In such cases, thorough patient counseling is essential to support informed decision‐making. Accordingly, LECS should be reserved for patients who are not suitable candidates for standard gastrectomy due to comorbidities, frailty, or personal preference.

With a median follow‐up period of 32 months, most deaths were non‐cancer‐related, while disease‐specific mortality occurred in two patients with advanced disease (pT4aN3 and pT1bN3). These outcomes likely reflected aggressive tumor biology, making it difficult to attribute cancer‐related deaths solely to LECS.

This study clearly defined LECS as a non‐curative, function‐preserving intervention. Patients were thoroughly informed about the treatment strategy, including the possibility of intraoperative detection of advanced disease. All participants consented in advance to complete local resection even in such scenarios, ensuring ethical integrity and respect for patient autonomy.

Importantly, no severe post‐gastrectomy syndromes (e.g., dumping and diarrhea) were observed. Body weight and muscle mass were maintained, and most patients remained physically and psychologically stable, suggesting a favorable impact on postoperative QOL. In clinical settings, high‐risk patients often receive no intervention, risking tumor‐related events such as bleeding, anemia, and obstruction, which can further impair QOL. LECS may therefore offer a meaningful balance between risk reduction and functional preservation in selected patients.

This study has several limitations. It was a single‐center, prospective study with a limited sample size, as enrollment did not reach the initially planned number. However, as this was an exploratory study focusing on the feasibility and clinical implications of a novel procedure, early publication of findings was considered valuable. Given that the safety profile was reasonably favorable, these results may serve as a foundation for future clinical research. Larger, multicenter studies are needed to validate the safety profile of LECS and define its optimal indications in gastric cancer treatment.

In conclusion, LECS appears to be a safe and feasible treatment option for selected elderly patients with gastric cancer who are not candidates for standard gastrectomy. Further studies are needed to confirm its long‐term oncologic safety and appropriate indications.

## Author Contributions

**Marie Washio:** conceptualization, data curation, formal analysis, funding acquisition, investigation, methodology, visualization, project administration, writing – original draft. **Keishi Yamashita:** conceptualization, formal analysis, supervision, writing – review and editing. **Takuya Wada:** conceptualization, data curation, investigation. **Mikiko Sakuraya:** data curation, investigation. **Hiroki Harada:** investigation. **Tadashi Higuchi:** investigation. **Koshi Kumagai:** investigation. **Yasushi Orihashi:** data curation, formal analysis. **Chika Kusano:** investigation, supervision, writing – review and editing. **Naoki Hiki:** conceptualization, investigation, methodology, project administration, supervision, writing – review and editing.

## Ethics Statement

Approval of the research protocol by an Institutional Reviewer Board: The protocol for this research project has been approved by a suitably constituted Ethics Committee of the institution and it conforms to the provisions of the Declaration of Helsinki. Committee of the Kitasato University School of Medicine. Approval No. C19‐303. Informed Consent: All informed consent was obtained from the subjects. Registry and the Registration No. of the study: UMIN Clinical Trials Registry (UMIN000039807). Animal Studies: Not applicable.

## Conflicts of Interest

The authors declare no conflicts of interest relevant to this article, except that H.H., K.Y., and N.H. serve as editorial board members of *Annals of Gastroenterological Surgery*.

## Supporting information


**Table S1:** Follow‐up schedule.

**Table S2:** QOL after LECS.
